# 3D Object Detection with SLS-Fusion Network in Foggy Weather Conditions

**DOI:** 10.3390/s21206711

**Published:** 2021-10-09

**Authors:** Nguyen Anh Minh Mai, Pierre Duthon, Louahdi Khoudour, Alain Crouzil, Sergio A. Velastin

**Affiliations:** 1 Cerema, Equipe-Projet STI, 1 Avenue du Colonel Roche, 31400 Toulouse, France; louahdi.khoudour@cerema.fr; 2 Institut de Recherche en Informatique de Toulouse, Université de Toulouse, UPS, 31062 Toulouse, France; alain.crouzil@irit.fr; 3 Cerema, Equipe-Projet STI, 8-10, Rue Bernard Palissy, 63017 Clermont-Ferrand, France; pierre.duthon@cerema.fr; 4 Department of Computer Science and Engineering, Universidad Carlos III de Madrid, Leganés, 28911 Madrid, Spain; sergio.velastin@ieee.org; 5 School of Electronic Engineering and Computer Science, Queen Mary University of London, London E1 4NS, UK

**Keywords:** adverse weather conditions, foggy perception, synthetic datasets, 3D object detection, autonomous vehicles

## Abstract

The role of sensors such as cameras or LiDAR (Light Detection and Ranging) is crucial for the environmental awareness of self-driving cars. However, the data collected from these sensors are subject to distortions in extreme weather conditions such as fog, rain, and snow. This issue could lead to many safety problems while operating a self-driving vehicle. The purpose of this study is to analyze the effects of fog on the detection of objects in driving scenes and then to propose methods for improvement. Collecting and processing data in adverse weather conditions is often more difficult than data in good weather conditions. Hence, a synthetic dataset that can simulate bad weather conditions is a good choice to validate a method, as it is simpler and more economical, before working with a real dataset. In this paper, we apply fog synthesis on the public KITTI dataset to generate the Multifog KITTI dataset for both images and point clouds. In terms of processing tasks, we test our previous 3D object detector based on LiDAR and camera, named the Spare LiDAR Stereo Fusion Network (SLS-Fusion), to see how it is affected by foggy weather conditions. We propose to train using both the original dataset and the augmented dataset to improve performance in foggy weather conditions while keeping good performance under normal conditions. We conducted experiments on the KITTI and the proposed Multifog KITTI datasets which show that, before any improvement, performance is reduced by 42.67% in 3D object detection for Moderate objects in foggy weather conditions. By using a specific strategy of training, the results significantly improved by 26.72% and keep performing quite well on the original dataset with a drop only of 8.23%. In summary, fog often causes the failure of 3D detection on driving scenes. By additional training with the augmented dataset, we significantly improve the performance of the proposed 3D object detection algorithm for self-driving cars in foggy weather conditions.

## 1. Introduction

Today, the trend is to bring automation applications into life to reduce the cost, human strength and improve working efficiency. While humans use sensory organs such as the eyes, ears, nose, and touch to perceive the world around them, industrial applications use sensors such as cameras, RADAR (radio detection and ranging), Kinect, LiDAR (light detection and ranging), IMU (inertial measurement unit) to collect data before being processed by complex algorithms. This research focuses mainly on self-driving cars, where cameras and LiDAR play a vital role in the perception of the environment. Cameras are often integrated in many practical computer vision applications such as retail [1], security [2], automotive industry [3], healthcare [4], agriculture [5], banking [6], and industrial automation [7]. Meanwhile, LiDAR is widely used to create high-resolution graphic products, with many advanced applications in the fields of geodesy [8], geomatics [9], archeology [10], geography [11], geology [12], geomorphology [13], mapping [14] and aeronautics [15]. This technology is also widely used for the sensing mechanisms of autonomous vehicles, and it is even integrated on Apple’s new product lines such as iPad Pro and iPhone 12 Pro as a 3D scanner. The fusion of cameras, LiDAR, and other sensors such as RADAR, GPS/IMU brings the ability to perceive the environment and then make operational decisions for self-driving cars.

**Figure 1:**
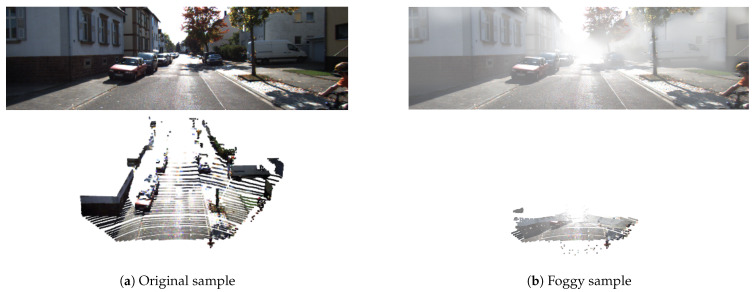
An example of distortion of an image and a point cloud in the proposed dataset. Note that the color of each point in the point cloud is the color of the corresponding pixel in the image (for visualization only). (**a**) shows the original sample from the KITTI dataset [16] with the image above and point clouds below collected by Velodyne HDL64 S2 LiDAR [16,17]. (**b**) shows the image and the point cloud simulated in foggy conditions with visibility equal to 52 m (*V* = 52 m) from the proposed dataset. Arising from receiving back-scattered light from water drops, fog makes the image have lower contrast and causes incorrect point measurements in the point cloud.

Autonomous vehicles have been put into tests or even commercial uses with varying degrees of automation from big tech companies such as Waymo, Uber, Lyft, Tesla, etc. However, these cars have not yet reached the level of performance in all circumstances and all weather conditions. Many factors can affect the perception ability of self-driving cars, thereby potentially causing serious consequences on the lives of other road users. One of the causes is that the data collected from these sensors are distorted due to environmental influences that may directly affect the awareness of self-driving cars. While applications using these sensors do quite well in controlled lighting or indoor environments unaffected by weather, outdoor applications face many problems. For example, applications that use a camera to perceive the environment often fail in extreme lighting conditions such as sunburns, low light, or nighttime conditions. Significant challenges arise in self-driving cars under adverse weather conditions such as fog, rain, or snow, in which both the camera and LiDAR are severely affected, as shown in Figure 1. Therefore, this work focuses on 3D object detection with camera and LiDAR sensors operating in foggy weather conditions.

Methods that produce the best results are mostly based on deep learning architectures by training a model with large amounts of data associated with labeling (supervised learning). While labeling and collecting data in good conditions such as daytime or sunny weather take time, it takes even more time and effort in extreme weather. Thus, there is an imbalance in the amount of data recorded in extreme weather conditions compared to those in normal conditions [18,19,20,21,22]. In addition, there may be an imbalance in different levels of fog, rain, or snow when collecting data. Most of the data are only collected during a given time and place, so the data will not be able to fully cover all situations, and therefore using a model trained with a limited range of data may make mistakes. So, in addition to real data, the creation of synthetic data which can be simulated under many controlled parameters is equally important. This paper aims at generating physics-based fog data on a well-known dataset collected in daytime and mild sunlight conditions to provide improvements under foggy conditions.

Some studies also identify failures and how to fix them in extreme weather conditions [23,24,25,26,27]. However, most of them only experiment on images and not on both images and point clouds [27]. To improve the performance of the perception algorithms, several studies suggest to dehaze fog, rain, or snow on images [23,28,29] and more recently for point clouds [30]. Others pointed out how the combination of LiDAR and cameras also affects performance [25,27]. Other studies proposed to increase the amount of data during the training phase [23,24,26,31].

This paper extends our previous work [32], a 3D object detector for self-driving cars in normal weather conditions, to run in foggy conditions. We point out that the late-fusion-based architecture can perform well with a justifiable training strategy in foggy weather conditions. The main contributions of this paper are:

- Firstly, we propose a new public dataset augmented from the KITTI dataset [16] for both images and point clouds through different visibility ranges (in fog) from 20 to 80 m to be as close as possible to a realistic foggy environment.
- Secondly, we show that the data collected from the camera and LiDAR are significantly distorted under foggy scenes. It directly affects the performance of self-driving cars 3D object detection algorithm, as confirmed through our experiments.
- Finally, extending from our previous work [32] on the original dataset [16] (good weather condition dataset), we propose a specific training strategy which uses both normal and foggy weather datasets as training datasets. Experiments show that the model can run better in foggy weather conditions while keeping performance close to that obtained in normal weather conditions.

The rest of this paper is organized as follows. A review of the related work is presented in Section 2. Section 3 describes the fog phenomenon, the fog rendering on camera and LiDAR. It also describes the 3D object detection method used in this paper. Numerical experiments and dataset generation are given in Section 4. Section 5 shows the results and gives its interpretation. Finally, we outline our conclusions and ongoing and future work in Section 6.

## 2. Related Work

This section aims to discuss and place the proposed work in the context of existing research. We distinguish between three main topics related to this work: Section 2.1 3D object detection, Section 2.2 datasets in adverse weather conditions and Section 2.3 perception in adverse weather conditions.

### 2.1. 3D Object Detection

Based on the sensors used as input, 3D object detection algorithms for self-driving cars are often divided into categories such as camera-based methods, LiDAR-based methods, and fusion-based methods. Since images do not provide depth information, methods of using RGB images [33,34,35,36] suffer from many difficulties and ambiguities when trying to predict the location of objects in 3D space. Mono3D [33] is the pioneering work on monocular 3D object detection. It is based on Fast RCNN [37], which is a popular 2D object detector plus many handcrafted features to predict 3D bounding boxes. These methods [35,36] try to predict 2D key points on the RGB image and then by combining with some constraint for each specific vehicle it can infer other points in 3D to get the final 3D bounding boxes. Pseudo-LiDAR [34] presents a new way of representing data. It generates a pseudo-point cloud by simply converting the predicted depth map from the image. Then it can leverage any LiDAR-based method for detection on the pseudo point cloud. In contrast, LiDAR provides the 3D point cloud data from which very accurate depth information from ego-vehicle to objects can be obtained. LiDAR-based methods [38,39,40,41,42] usually give very good results for perception tasks and have received much attention in recent years. PointRCNN [38] is a two stage detector which attempts to extend Faster RCNN [43] and Mask RCNN [44] to the point cloud representation. VoxelNet [40] and PointPillars [42] try to encode the point cloud into 3D cells (voxel or pillar), similar to images but with a height channel, and then use the 3D CNN to extract features. Meanwhile, methods [45,46,47] that attempt to combine both images and point cloud have yet to really stand out from the LiDAR methods, despite taking in both streams of information. F-PointNet [46] or F-ConvNet [47] firstly use the 2D bounding box of objects detected from images to find the frustum region in 3D space. Then, PointNet segmentation [48] is used to find objects in each frustum. MV3D [45] uses point cloud to generate 3D proposal boxes, plus sensor fusion layers to refine final 3D bounding boxes. The point cloud is represented as images (bird’s eye view and front view). These methods are usually quite cumbersome and cannot yet run in real time.

### 2.2. Datasets in Adverse Weather Conditions

Most common existing datasets have been collected in good conditions such as KITTI [16], Cityscape [49], or in different lighting conditions such as BDD100K [19], Waymo [50], NuScenes [18]. More recent attention has focused on the perception ability of self-driving cars in adverse weather conditions, because such conditions negatively affect the quality of the camera and LiDAR sensing, resulting in degraded performance. Consequently, some datasets have been collected in foggy conditions including Foggy Driving [23], Foggy Zurich [31], SeeingThroughFog [27], nuscenes [18], BDD100k [19], Oxford dataset [20,21,22,51], rain [51,52] or snow [51,53,54] condition.

However, data collection under such conditions is not easy, and it may cause post-processing problems such as imbalance issues or labeling errors. In contrast, synthetic datasets are increasingly close to real data and can avoid such problems. Synthetic datasets can be divided into two categories: physics-based such as Foggy Cityscapes [23], RainCityscapes [55], Foggy Cityscapes [31], Rain augmented [24,56,57] and generative adversarial network based (GAN-based) such as [56,58].

Despite the usefulness of these datasets above, KITTI dataset [16] is commonly used in the literature, and it’s easy to work on. We decide to use this dataset as a base dataset for further fog rendering on it. While most synthetic datasets focus only on images [23,24,31,55,56], this work aims to take into account fog for both image and point cloud starting from a good weather dataset [16]. We use the physics-based procedure proposed in [27] to retain physical properties like real fog.

### 2.3. Perception in Adverse Weather Conditions

While outdoor perception algorithms are usually more sensitive under varying lighting conditions than indoors [59,60], perception under extreme weather conditions is even more challenging [23,24,27] because of sensor degradation, lower contrast, limited visibility and thereby causing errors in the prediction. In fact, previous research has shown how performance drops in segmentation [23,24,25,26,31], 2D object detection [23,26,27] and depth estimation [26]. Some studies have also shown that performance may be improved by learning with synthetic data [23,24,26,31], dehazing [23] or using late-based fusion [25,27].

Like previous works [23–27,31], this research aims to analyze the perception algorithms of self-driving cars under foggy scenes, with a special emphasis on the 3D object detection task. Indeed, performance is greatly reduced for 3D object detection in foggy scenes but by training both with a normal and a synthetic dataset, performance may be improved. Overall, these findings are consistent with those reported in [23,24,26,31].

## 3. Methods

This section presents the definition of the fog phenomenon, how fog is modeled to generate the proposed Multifog KITTI dataset, mentioned later in Section 4. Then, we extend our previous 3D object detection work [32] to investigate its performance under foggy weather conditions.

### 3.1. Fog Phenomenon

Fog is the phenomenon of water vapor condensing into tiny cloud-like particles that appear on and near the ground instead of in the sky. The Earth’s moisture slowly evaporates, and when it does, it moves up, cools, and condenses to form fog. Fog can be seen as a form of low clouds.

**Figure 2:**
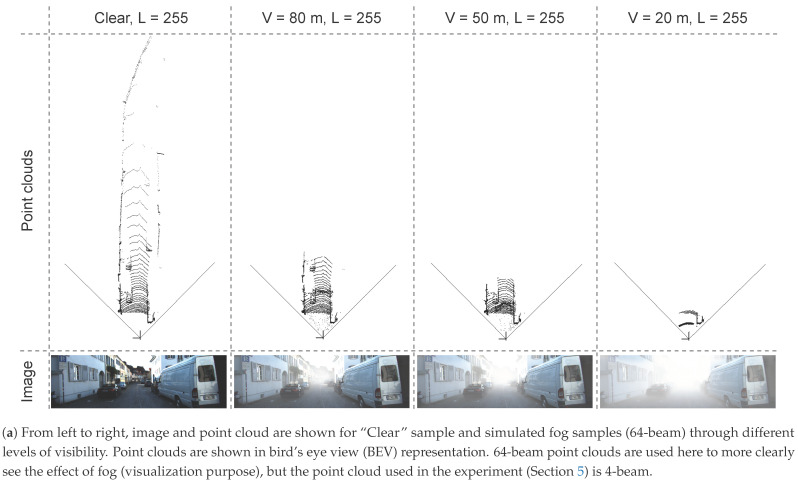

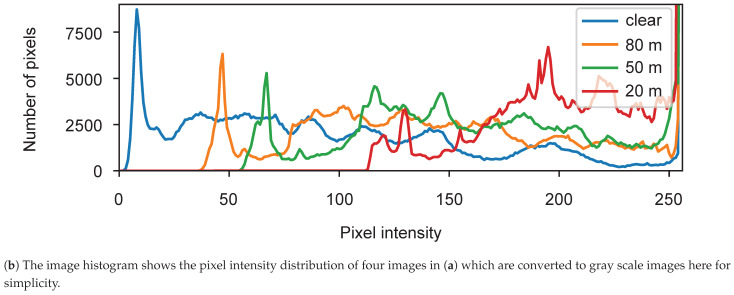
The distortion of the image and point cloud according to different levels of visibility in a foggy scene. As visibility decreases, the contrast of the image and the range of the point cloud also decrease significantly, as shown in (**a**,**b**).

Physically, fog is a phenomenon that causes dispersion. Light is scattered by the suspended water droplets before falling into the image sensor. This scattering phenomenon has two primary effects. First, the chief ray is attenuated before falling into the sensor, and second, a signal floor of scattering light is present. These effects reduce contrast, as shown in Figure 2, the intensity range is filled with intensity values (a single value for a gray-level image for each pixel) that decrease with the intensity of the fog (clear, 80, 50, 20 m). Therefore, the contrast of the image is inversely proportional to fog density, and it may cause driving difficulties both for human drivers and sensor-based autonomous systems or driving aids.

The meteorological optical range (MOR), also called visibility and denoted by *V*, is the distance in meters where contrast is no longer distinguishable on a white and black target. A white and black target is perceived by the human eye as uniformly gray when moving away in the fog. A limit contrast level of 5% has been defined in the standard [61]. The lower the MOR, the denser the fog. By international agreement, the term fog is used when visibility *V* is less than 1 km [62].

### 3.2. Fog Rendering

Datasets under extreme weather conditions are numerically less numerous than those under normal conditions (clear weather). Firstly, adverse weather conditions do not occur frequently. Secondly, it is more difficult to clean and label this kind of data. It causes an imbalance problem for real datasets between different types of weather conditions. Therefore, the generation of synthetic datasets is useful to develop a system that works in adverse weather conditions.

There are different ways to generate artificial data under foggy weather conditions: (a) acquisition under controlled conditions [63] or (b) augmentation on a normal condition dataset. For the second type, there are also different ways to model fog, such as physics-based [23,24,31,55,56] or GAN-based [56] modeling. In this paper, we use the physics-based method [23,27,64] as it can keep the physical properties of the weather, and it has been researched for a long time.

While radar is not significantly affected by fog [65], data collected from LiDAR and cameras are quite distorted as shown in Figure 1 and. The following describes how point clouds (LiDAR) and images (camera) are modeled and tested later to show the influence of fog on these sensors and the performance of 3D object detection algorithms.

#### 3.2.1. Camera in Fog

Based on Koschmieder Law [64] in 1924, Sakaridis et al. [23] formulated the equation to obtain an observed foggy image $I_{foggy}\left(u,v\right)$ at pixel (u,v) as follows:

(1) $$I_{foggy}\left(u,v\right)=t\left(u,v\right)I_{clear}\left(u,v\right)+\left(1-t\left(u,v\right)\right)L,$$

where $I_{clear}\left(u,v\right)$ denotes a latent clear image, *L* the atmospheric light which is assumed to be globally constant (generally valid only for daytime images), and in case of a homogeneous medium, the transmission coefficient is:

(2) t(u,v)=exp(−βD(u,v)),

where β is the fog density (or attenuation) coefficient, and D(u,v) is the scene depth at pixel (u,v).

Fog thickness is controlled by β. Using Koschmieder’s Law, visibility *V*, can be described by equation [61] (page I-9.4):

(3) $$C_{T}=exp\left(-\beta V\right)$$

or,

(4) $$V=-\frac{ln(C_{T})}{\beta },$$

where $C_{T}$ is the contrast threshold. According to the International Commission on Illumination (CIE) [66], $C_{T}$ has a value of 0.05 for daytime visibility estimation. We can also express t(u,v) as a dependency on visibility *V* and the depth map *D* as follows:

(5) $$t\left(u,v\right)=exp\left(\frac{ln\left(C_{T}\right)D\left(u,v\right)}{V}\right).$$

#### 3.2.2. LiDAR in Fog

Gruber et al. [27] assume that beam divergence is not affected by fog. In this model, a returned pulse echo is always registered as long as the received laser intensity is larger than the effective noise floor. However, severe back-scatter from fog may lead to direct back-scatter from points within the scattering fog volume, which is quantified by the transmissivity t(u,v). Then, the observed foggy LiDAR $L_{foggy}\left(u,v\right)$ can be modeled using the following equation [27]:

(6) $$L_{foggy}\left(u,v\right)=t\left(u,v\right)L_{clear}\left(u,v\right),$$

where $L_{clear}\left(u,v\right)$ and $L_{foggy}\left(u,v\right)$ are the intensity of the light pulses emitted from LiDAR and the received signal intensity, respectively.

Modern scanning LiDAR systems implement adaptive laser gain to increase the signal for a given noise floor, resulting in the maximum distance:

(7) $$d_{max}=\frac{1}{2\beta }ln\left(\frac{n}{L_{clear}+g}\right),$$

where *n* is the detectable noise floor.

This section has shown how to digitally add fog to a dataset initially acquired in clear weather. The 3D object detection method applied to this dataset is presented next.

### 3.3. 3D Object Detection Algorithm

**Figure 3:**
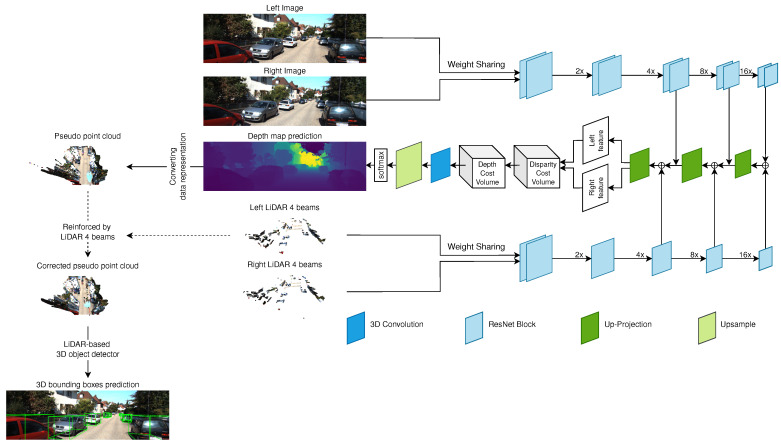
Overview of the SLS-Fusion framework.

Here, we use our previous 3D object detection algorithm, called SLS-Fusion [32] and inspired by the work of Qiu et al. [67]. Figure 3, shows a block diagram for this 3D detector. It takes a pair of stereo images and the re-projected depth map of simulated 4-beam LiDAR on the left and right image as input. It uses late-fusion and is divided into 3 parts: depth estimation, converting data representation, and LiDAR-based 3D object detection.

The model takes stereo images ($I_{l}$, $I_{r}$) and the corresponding simulated stereo images by projecting 4-beam LiDAR, for the left and right side ($S_{l}$, $S_{r}$). $S_{l}$ and $S_{r}$ are simulated using the formula proposed in [68]. An encoder-decoder network is used to extract features from both images and point clouds. The proposed network has a weight-sharing pipeline for both LiDAR and images, ($I_{l}$, $S_{l}$) and ($I_{r}$, $S_{r}$), instead of only using left and right images as proposed in [68,69]. Once the left and right features are obtained from the decoding stage, they are passed to the Depth Cost Volume (DeCV) proposed in [68] to learn the depth information. Here, as in [68], the smooth L1loss function is used:

(8) $$\sum_{\left(u,v\right)\in I_{l}}^{}\left|d(u,v)-D(u,v)\right|,$$

where d(u,v) denotes the valid depth ground truth. The predicted depth map is *D*, where D(u,v) is the depth corresponding to the pixel (u,v) in the left image $I_{l}$. Then, pseudo point clouds are generated using a pinhole camera model. Given the depth D(u,v) and camera intrinsic matrix, the 3D location ($X_{c}$, $Y_{c}$, $Z_{c}$) in the camera coordinate system for each pixel (u,v) is given by:

(9a) $$\begin{array}{cc} \left(\mathrm{depth}\right)Z_{c} & =D(u,v) \end{array}$$

(9b) $$\begin{array}{cc} \left(\mathrm{width}\right)X_{c} & =\frac{\left(u-c_{U}\right)\times Z_{c}}{f_{U}} \end{array}$$

(9c) $$\begin{array}{cc} \left(\mathrm{height}\right)Y_{c} & =\frac{\left(v-c_{V}\right)\times Z_{c}}{f_{V}} \end{array}$$

where $c_{U}$ and $c_{V}$ are the coordinates of the principal point and $f_{U}$ and $f_{V}$ are respectively the focal length in pixel width and height. Following [68], 4-beam LiDAR are used to reinforce the quality of the pseudo point cloud. Then each point ($X_{c}$, $Y_{c}$, $Z_{c},1$) is transformed into ($X_{l}$, $Y_{l}$, $Z_{l},1$) in the LiDAR coordinate system (the real world coordinate system). The pseudo point cloud is filled by adding reflectance as 1. Given the camera extrinsic matrix $C=\left[\begin{array}{cc} R & t\\ 0 & 1 \end{array}\right]$, where *R* and *t* are respectively the rotation matrix and translation vector. The pseudo point cloud can be obtained as follows:

(10) $$\begin{array}{c} \left[\begin{array}{c} X_{l}\\ Y_{l}\\ Z_{l}\\ 1 \end{array}\right]=C^{-1}\left[\begin{array}{c} X_{c}\\ Y_{c}\\ Z_{c}\\ 1 \end{array}\right]. \end{array}$$

Once the pseudo point cloud is obtained, it can be treated like a normal point cloud, although its accuracy depends on the quality of the predicted depth. Similar to Pseudo-LiDAR++ [68], the input (4-beam point clouds) is used to correct for errors in the pseudo point cloud. This is a refinement step to obtain a more accurate point cloud. Then, the depth map is converted into a pseudo point cloud. The idea is to leverage the performance of current leading LiDAR-based methods such as PointRCNN [38] to detect objects.

## 4. Experiments

### 4.1. Datasets and Evaluation Metrics

#### 4.1.1. Datasets

This part presents the datasets that have been used for this work, and it introduces a new synthetic dataset in foggy situations for both image and LiDAR-based on the KITTI dataset [16]. As the detector [32] is based on the pseudo-LiDAR pipeline [34], the training procedure is divided into two parts: depth prediction and 3D object detection. The corresponding dataset for each part is introduced as follows. The Scene Flow dataset [70], a large-scale synthetic dataset, is used first for training the depth estimation part. It has 35,454 images of size 960 × 540 for training and 4370 images for testing.

The KITTI object detection dataset [16] is then used for both fine-tuning the depth estimation part and for training the 3D object detection part. To avoid confusion, from now it is referred to as Clear KITTI dataset. It is the most common dataset for driving scenes collected in the daytime and under good weather conditions. It has 7481 training samples and 7518 testing samples for both stereo and LiDAR. Like most other studies, here the training dataset is divided into a training part (3712 samples) and a validation part (3769 samples) as proposed in [45].

**Figure 4:**
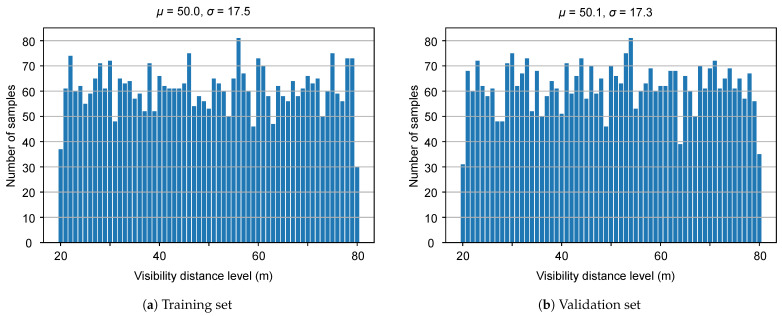
Distribution of MOR of different parts in the Multifog dataset.

The proposed Multifog KITTI dataset is generated by using the equations given in Section 3 for different visibility levels from 20 to 80 m. As the depth map is required in Equation (5) to calculate the transmission coefficient t(u,v) and then $I_{foggy}\left(u,v\right)$ in Equation (1) and $L_{foggy}\left(u,v\right)$ in Equation (6) for each frame, the algorithm proposed in [71] is applied to the depth map *D* corresponding to each image on the left side $I_{l}$ and the same for the right image $I_{r}$. This method takes an RGB image and a sparse depth image as input and results in an image where the value of each pixel is the depth information. The default configuration proposed in [27] is used, with g=0.35 and n=0.05 for the Velodyne HDL64 S2 LiDAR used in the KITTI dataset. Figure 4 shows the number of samples for each visibility level for the training and validation sets of the Multifog KITTI dataset. This dataset is used similarly to the Clear KITTI dataset for both depth estimation and 3D object detection parts. The proposed Multifog KITTI dataset contains 7481 training samples and 7518 testing samples for stereo images, 64-beam LiDAR, and 4-beam LiDAR. The 64-beam LiDAR data were not used in this work.

#### 4.1.2. Evaluation Metrics

To measure the performance of the 3D object detection task, average precision (AP) is computed across 11 recall positions values between 0 and 1 as proposed in [72] for both 3D and bird’s eye view (BEV) levels with intersection over union (IoU) thresholds at 0.5 and 0.7.

According to [16], objects are divided into three levels of difficulty: Easy, Moderate and Hard, depending on the 2D bounding box sizes, occlusion, and truncation extent appearing on the image. This study focuses on detecting “Car” objects because the car is one of the main objects and occupies the largest percentage in the KITTI dataset.

### 4.2. Experimental Protocols

To clearly present the different steps of the experiments, this part presents the list of experiments carried out and explains why they were undertaken.

As said earlier, for 3D object detection, SLS-Fusion [32] was used. Satisfactory results have been achieved with this algorithm on the KITTI dataset. To verify whether the model is still satisfactory when dealing with foggy weather conditions, the model which is trained on Clear KITTI is evaluated on MultiFog KITTI.

Secondly, the opposite of the above test is tested. The model is trained on the Multifog KITTI and then evaluated on Clear KITTI and Multifog KITTI dataset. The purpose of this experiment and the one above is to verify whether the model performance is affected by swapping the training and the evaluation datasets.

Then, we train on both Multifog and Clear KITTI together and then evaluate on each dataset to see whether performance is improved.

Finally, the proposed algorithm is compared with the leading low-cost sensors based method, Pseudo-LiDAR++ [68] which uses both images and 4-beam LiDAR as input. The results are presented on the Clear KITTI and the Multifog KITTI datasets.

### 4.3. Implementation Details

Both training and evaluation processes are carried out similarly through the experiments presented in Section 4.2.

The 4-beam LiDAR used in the input of the network is simulated based on the HDL-64E LiDAR sensor to resemble the ScaLa sensor as proposed in [68].

For both training and evaluation, the depth estimation part, which is written in the pytorch framework, is trained and evaluated first, on the Scene Flow dataset. As Scene Flow does not contain point cloud data, the input LiDAR is set to zeros. Then the network is fine-tuned on the KITTI dataset (Clear or Multifog) for 100 epochs with a batch size of 4 and a learning rate of 0.001. For the detector part, the common LiDAR-based object detector PointRCNN [38] is employed using the default configuration for both training and evaluation. PointRCNN is a LiDAR-based method with high performance and used by many other methods and used in this work to detect objects based on point clouds. As it was designed to take into account sparse point clouds, the dense point cloud is sub-sampled to 64-beam LiDAR. Then, the released implementations of PointRCNN is used directly, and their guidelines are followed to train it on the training set of the KITTI object detection dataset. Like our previous work, we only train for the “Car” class because car is one of the main objects and occupies the largest percentage in KITTI dataset, which causes the imbalance between “Car” and other classes, as solving this imbalance problem is not the goal of this work.

All experiments in this paper were run on 2 NVIDIA GeForce GTX 1080 Ti GPUs with 11 GB memory and on the Ubuntu operating system.

## 5. Results

As shown in Table 1 and Table 2, the results of different tests using our SLS-Fusion method are respectively shown for IoU (Intersection over Union) of 0.5 and IoU of 0.7. In each cell of these tables, a pair of numbers A/B corresponds to the results obtained with the $AP_{BEV}$ and $AP_{3D}$ metrics on the Clear KITTI or Multifog KITTI datasets (Table 1 for IoU of 0.5 and Table 2 for IoU of 0.7). These two tables show the results and methods achieved through different experiments on the training dataset and the testing dataset. Similarly, Table 3 and Table 4 compare SLS-Fusion [32] with Pseudo-LiDAR++ [68].

### 5.1. SLS-Fusion Algorithm Evaluation in Foggy Conditions

In this section, the SLS-Fusion 3D object detector is evaluated for different cases of training data and test data, and the results are reported in Table 1 (IoU = 0.5) and Table 2 (IoU = 0.7). In both tables, the first row shows the detection results for our SLS-Fusion method [32] when the network is trained and evaluated on the Clear KITTI dataset.

The following row (number 2 in both tables) shows that the detection performance is drastically reduced when using weights trained on Clear KITTI and then evaluated on Multifog KITTI (foggy dataset). This shows that the method, trained only on clear weather datasets, does not respond well to degraded conditions. Note that these data are not dehazed before it is put to the model.

Subsequently, the objective is to investigate the detection performance when under fog (row number 3). The training part of the Multifog KITTI dataset is used as training data, and the test part of Multifog KITTI is used for testing. It shows that detection performance increases markedly whether, for the Easy, Moderate, or Hard cases compared to the Clear/MultiFog test (row number 2).

We now want to check that the weights trained for the Multifog KITTI (row number 3) remain relevant in the case of clear conditions. For this, we test it on Clear KITTI dataset (row number 4). The results are quite poor, which is understandable because the dataset in testing (Clear) is different compared to the one in training (Multifog) (similar to the Clear/ Multifog test). This shows that the training dataset must clearly be adapted to all meteorological conditions, whether good or bad.

Finally, the last tests (rows 6 and 7 of the two tables) consist in using training datasets as broadly as possible. In this case, we used the Multifog and Clear KITTI datasets for training, and tested on the Clear and Multifog datasets independently. In general, it was found that the results were better compared to the experiments trained on the individual datasets (either Clear KITTI or Multifog KITTI). A trade-off between the performance on both datasets (Clear and Multifog KITTI) and the number of training times can also be made.

**Figure 5:**
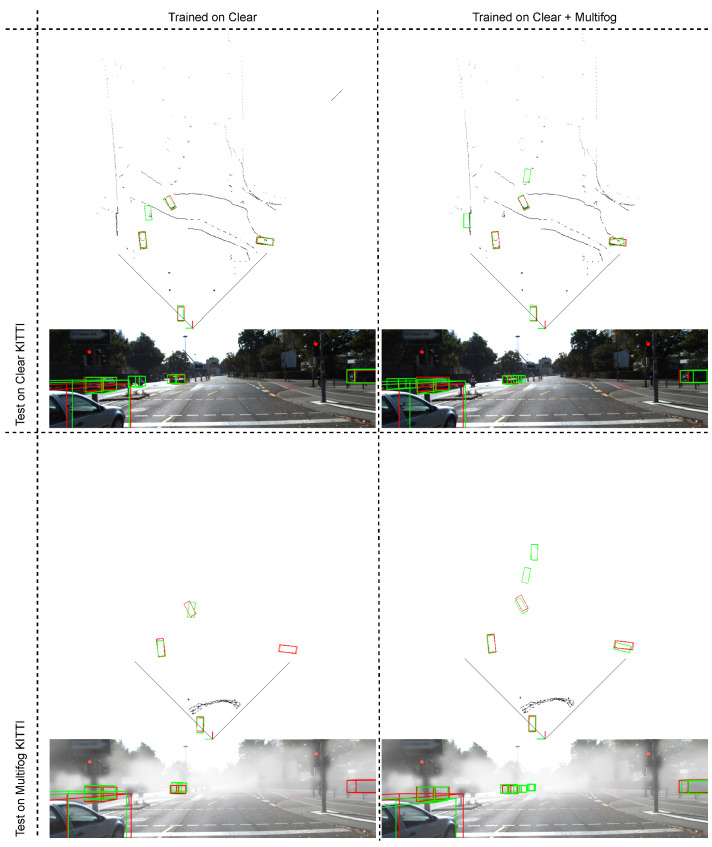
Qualitative results of our SLS-Fusion method for 3D object detection on the Clear KITTI dataset and the proposed dataset (Multifog KITTI). 3D bounding boxes in red and in green denote the ground truth and the prediction for objects in the scene, respectively. Note that the point cloud is shown in BEV representation.

Consequently, a way to correctly address the problem of fog conditions was found, which was initially very problematic (extremely low scores on row number 2) by adapting the training process. We have shown that this training must take into account all weather conditions to be effective. Finally, we managed to maintain performance under normal conditions, which is very positive. Figure 5 presents some illustrative results of car detection, showing 3D predicted bounding boxes. It is consistent with the results reported above that training on Clear and Multifog gives better results.

In the next part, our SLS-Fusion method is compared with the leading method for low cost based 3D object detection method, Pseudo-LiDAR++ [68].

### 5.2. Comparison with Pseudo-LiDAR++ Method

Here, the SLS-Fusion method is compared with the leading method Pseudo-LiDAR++ (images and+ 4-beam LiDAR as inputs) on Clear KITTI dataset (normal weather conditions scenes). It was found that SLS-Fusion provided better results on several metrics, as shown in Table 3 and Table 4. Better results are in bold-italic. These tables, have all the comparisons ($AP_{BEV}$/$AP_{3D}$) for IoU = 0.5 (Table 3) and IoU = 0.7 (Table 4).

In Table 3 (IoU = 0.5), rows 1 and 2 show results reported at [32] for the experiment on Clear KITTI. It can be noticed that SLS-Fusion is better in some categories, such as $AP_{3D}$ for Easy level objects (93.02% against 90.3%). For Moderate level objects, the algorithm achieves 88.8% against 87.7% ($AP_{BEV}$). The results are similar for Table 4 (IoU = 0.7) where the results are better for Easy and Moderate level objects such as $AP_{3D}$ for Easy level objects (76.7% against 75.1%), $AP_{3D}$ for Moderate (63.9% against 63.8%) and slightly worse for Hard level objects. SLS-Fusion, tends to achieve better results for Easy and Moderate level objects which are closer and less overlapped. This is explained by the backbone of SLS-Fusion which fuses both features from images and the 4-beam point cloud, while Pseudo-LiDAR++ uses only images as input for feature extraction. However, 4-beam LiDAR is very sparse and has almost no points for distant or occlusion objects, its contribution is almost non-existent while the number of parameters in the model is still more. This leads to lower results for Hard level objects.

For the experiment on Multifog KITTI, Table 3 (rows 3 and 4) shows that the SLS-Fusion algorithm outperforms Pseudo-LiDAR++ in all comparisons, regardless of the object difficulty. For example, for 3D detection comparison ($AP_{3D}$), the method achieved 89.82% versus 88.65% for Easy, 78.19% versus 77.21% for Moderate and 75.05% versus 69.59% for Hard level objects. It indicates that the SLS-Fusion model is better trained when the both Clear and Multifog KITTI are used. The extraction step of SLS-Fusion is better than Pseudo-LiDAR++. However, in Table 4 (rows 3 and 4), it can be seen that the algorithm is still better on the $AP_{BEV}$ metric for every level of difficulty objects such as 84.30% against 82.47% for Easy, 63.12% $AP_{BEV}$ against 62.54% for Moderate or 57.84% against 56.87% for Hard, but it is worse on the $AP_{3D}$ metric compared to Pseudo-LiDAR++ method. This IoU level (0.7) is more difficult in terms of localization, so our method does not produce superior results.

## 6. Conclusions

The main objective of this research was to analyze the effect of fog on 3D object detection algorithms. To do this, a novel synthetic dataset was created for foggy driving scenes. It is called Multifog KITTI dataset. This dataset was generated from the original dataset, KITTI dataset, by applying fog at different levels of visibility (20 to 80 m). This dataset covers the left and right images, 4-beam LiDAR data and 64-beam LiDAR data, although the foggy 64-beam data are yet to be exploited.

This work found that the addition of fog to the images and the LiDAR data leads to irregularities (lower contrast) in the images and distortions in the 3D point clouds of the LiDAR targets. The objective was then to test the robustness of the SLS-Fusion algorithm in dealing with the degradation of image and LiDAR data. The first list of tests consisted in verifying the negative effect of fog on the detection algorithm. Processing foggy data as input and using normal data in training leads to a degradation of results. Thus, the degradation of the detection of 3D objects is observed (detection rate decreasing from 63.90% to 21.23% for Moderate level objects). The second major finding was that the performance of the 3D object detection algorithm can be improved by directly training with both the KITTI dataset and the synthetic Multifog KITTI dataset, even without dehazing. These results add to the rapidly expanding field of perception in adverse weather conditions, specifically in foggy scenes.

Another test consisted in comparing our SLS-Fusion algorithm with the leading low-cost sensors based method, which is Pseudo-LiDAR++. It was discovered that our method outperforms the Pseudo-LiDAR++ method on different metrics for the proposed Multifog KITTI dataset. This result is very satisfactory because it shows the robustness of the method when dealing with foggy datasets.

The scope of this study was limited in terms of the effect of point clouds. As the data used in the experiments are 4-beam point clouds, its features are not as rich as the features from images or 64-beam point clouds. These findings provide the following insights for future research: testing 64-beam LiDAR-based 3D objects detection algorithms on the Multifog KITTI dataset to show more clearly the effects of the fog on point clouds. We also plan to deal with the case of when either cameras or LiDAR are damaged due to the influence of the weather. 

## Figures and Tables

**Table 1:** $AP_{BEV}$/$AP_{3D}$ results with training and testing on the Clear KITTI or Multifog KITTI datasets for “Car” objects with IoU = 0.5 on three levels of difficulty: Easy, Moderate and Hard.

Method	Training Set	Test Set	${AP}_{BEV}/{AP}_{3D}@0.5$		
			Easy	Moderate	Hard
Our	Clear	Clear	93.16/93.02	88.81/86.19	83.35/84.02
Our	Clear	MultiFog	72.66/66.60	47.11/41.86	41.24/37.97
Our	MultiFog	MultiFog	90.27/90.15	79.17/78.01	76.12/70.21
Our	MultiFog	Clear	57.33/55.00	39.10/38.38	33.55/32.58
Our	MultiFog + Clear	Clear	95.36/95.10	86.90/85.04	84.75/79.10
Our	MultiFog + Clear	MultiFog	89.94/89.82	79.13/78.19	76.36/75.05

**Table 2:** ${AP}_{BEV}$/ ${AP}_{3D}$ results with training and testing on the Clear KITTI or Multifog KITTI datasets for “Car” objects with IoU = 0.7 on three levels of difficulty: Easy, Moderate and Hard.

Method	Training Set	Test Set	${AP}_{BEV}/{AP}_{3D}@0.7$		
			Easy	Moderate	Hard
Our	Clear	Clear	87.51/76.67	76.88/63.90	73.55/56.78
Our	Clear	MultiFog	44.57/30.89	29.76/21.23	26.50/18.61
Our	MultiFog	MultiFog	83.42/69.57	62.79/48.19	56.84/44.85
Our	MultiFog	Clear	41.54/30.07	27.74/21.10	23.87/18.37
Our	MultiFog + Clear	Clear	86.23/72.14	72.15/55.67	67.40/53.57
Our	MultiFog + Clear	MultiFog	84.30/69.07	63.12/47.95	57.84/45.50

**Table 3:** Comparison with Pseudo-LiDAR++ [68] method, with IoU = 0.5, best results shown in bold-italic.

Method	Training Set	Test Set	${AP}_{BEV}/{AP}_{3D}@0.5$		
			Easy	Moderate	Hard
PL++	Clear	Clear	90.3/90.3	87.7/**86.9**	**84.6**/**84.2**
Our	Clear	Clear	**93.16**/**93.02**	**88.81**/86.19	83.35/84.02
PL++	MultiFog + Clear	MultiFog	88.77/88.65	78.39/77.21	75.23/69.59
Our	MultiFog + Clear	MultiFog	**89.94**/**89.82**	**79.13**/**78.19**	**76.36**/**75.05**

**Table 4:** Comparison with Pseudo-LiDAR++ [68] method, with IoU = 0.7, best results shown in bold-italic.

Method	Training Set	Test Set	${AP}_{BEV}/{AP}_{3D}@0.7$		
			Easy	Moderate	Hard
PL++	Clear	Clear	**88.2**/75.1	**76.9**/63.8	73.4/**57.4**
Our	Clear	Clear	87.51/**76.67**	76.88/ **63.90**	**73.55**/56.78
PL++	MultiFog + Clear	MultiFog	82.47/**70.22**	62.54/**48.34**	56.87/**45.74**
Our	MultiFog + Clear	MultiFog	**84.30**/69.07	**63.12**/47.95	**57.84**/45.50

## Data Availability

Data available on request from the authors or from https://github.com/maiminh1996/SLS-Fusion (accessed on 4 October 2021).

## Abbreviations

The following abbreviations are used in this manuscript:

AP	Average precision
BEV	Bird’s-eye-view
IoU	Intersection over Union
LiDAR	Light Detection and Ranging
MOR	Meteorological Optical Range
PL	Pseudo-LiDAR
PL++	Pseudo-LiDAR++
SLS-Fusion	Sparse LiDAR and Stereo Fusion
